# The National Insurance (Navy and Army) Act, 1914

**Published:** 1914-09-19

**Authors:** 


### THE NATIONAL INSURANCE (NAVY AND ARMY) ACT, 1914.

### The Position of Soldiers for Lord Kitchener's Armies.

The enlistment of soldiers in Lord Kitchener's armies for the present war has necessitated the introduction into Parliament of a short Bill to amend the provisions of the National Insurance Act of 1911 in respect of them. It is proposed in this measure, in effect, that all soldiers specially enlisting for the purposes of the war shall be treated in regard to National Health Insurance as though they were members of the Regular Army. That is to say, the only benefit to which they will be entitled from the Insurance funds while they are serving with the Colours will be maternity benefit, while the weekly contribution deducted from their pay will be 1½d. instead of the 4d. normally applicable to male employed contributors. Most of the men so enlisting will doubtless already be members of approved societies, and as such they will continue to receive the maternity benefit from their individual societies. Their societies will, in turn, obtain the appropriate proportion of their contributions. Those who are not already members of societies may elect to join societies, if they so desire, or, if they fail to do so, the Navy and Army Insurance Fund will receive the contributions and pay the benefit.

The weekly contribution is more than sufficient to provide the maternity benefit, and carries a proportion which is applied to enable the soldier to receive normal benefits on his discharge. By this means a soldier enlisting for the war is secured the right to maternity benefit, if his wife is confined while he is on service, and he will not suffer on re-entry into full insurance when he eventually leaves the Army. It may be added that approved societies, at the request of the Insurance Commissioners, are taking steps to see that there is no unnecessary delay in the payment of maternity benefit owing to the absence of the husband. The Bill goes on to provide that the special provision of the National Insurance Act shall apply "to all persons who, being previously insured, serve during the present war as commissioned or warrant officers of the Naval Reserves, or officers of the Reserve, or of the Territorial Force, or are granted temporary commissions in the regular forces during the continuance of the present war, as it applies to men of the Territorial Forces called out on embodiment."

This is only an extension of what has already been said in regard to soldiers. There is, however, this distinction—namely, that in the case of the officers of the descriptions cited the provisions of the Act are only to apply when they were previously insured. In other words, the fact that the pay while on service is below the limit of £160 a year is not of itself to render it necessary to become an insured person.

The Bill appears to meet the situation satisfactorily, as its outcome will be that all those enlisting who are already insured persons will have secured for them while on service the only benefit that is of any practical value during their absence from home, and will, moreover, maintain the full privileges of their insurance on return to civil employment. Those joining the ranks who are not already insured will become insured, while in the case of officers contributions will only be deducted from their pay when they are already insured.

